# The clinical characteristics and diagnostic and treatment protocol for 14 acute *Vibrio vulnificus* infections caused by aquatic products

**DOI:** 10.1097/MD.0000000000043942

**Published:** 2025-08-22

**Authors:** Donghua Ma, Jinjun Wang, Baoying Fan, Jianji Liang, Qing Liu, Zhiyong He

**Affiliations:** a Department of Orthopaedics, Zhongshan City People’s Hospital, Zhongshan, China; b College of General Practice, Southern University of Science and Technology, Shenzhen, Guangdong, China.

**Keywords:** aquatic products, diagnostic and treatment protocol, infection, mNGS, *Vibrio vulnificus*

## Abstract

*Vibrio vulnificus* infections caused by aquatic product-related injuries pose severe clinical challenges due to their rapid progression and high morbidity and mortality. Early diagnosis and timely intervention are critical to improving patient outcomes, yet standardized diagnostic and treatment protocols remain limited. We conducted a retrospective descriptive case series of 14 patients with confirmed *V vulnificus* infection admitted between 2020 and 2023. Clinical data, including demographic characteristics, injury history, symptoms, laboratory results, diagnostic methods, treatment strategies, and outcomes, were collected and analyzed. Microbial culture and metagenomic next-generation sequencing (mNGS) were compared in terms of diagnostic timing. All 14 patients had a clear history of aquatic product-related trauma, with 85.7% (12/14) presenting within 24 hours of injury. The average time from injury to symptom onset was 13.11 ± 6.61 hours. All patients exhibited limb erythema, swelling, warmth, and pain; 11 patients (78.6%) developed complications such as sepsis (42.9%), compartment syndrome (35.7%), or multiple organ dysfunction syndrome (28.6%). Ten cases were confirmed by microbial culture (average time: 1.68 ± 0.63 days), and 4 by mNGS (average time: 1.00 day). The average time to diagnosis was shorter in patients diagnosed by culture than those requiring mNGS (1.86 ± 0.68 vs 4.82 ± 0.90 days). All patients received empirical combination antibiotic therapy upon admission; the average duration of intravenous antibiotic treatment was approximately 10 days. Six patients (42.9%) underwent amputation, and 2 (14.3%) died. Among the survivors, 50% achieved Brunnstrom stage V to VI hand function recovery at final follow-up. Early identification and aggressive treatment are essential in managing *V vulnificus* infections related to aquatic product injuries. mNGS plays an important supplementary role in diagnosis, especially in culture-negative cases. The proposed diagnostic and treatment protocol, based on real-world experience, may help improve clinical decision-making and reduce poor outcomes such as amputation and death.

## 1. Introduction

*Vibrio vulnificus* infections occur sporadically in clinical settings, with a relatively low overall incidence but a high mortality and disability rate.^[1–3]^ Research indicates that the pathogenicity of *V vulnificus* is associated with certain components of the bacterium, such as biofilms, capsule polysaccharides, hemolysins, lipopolysaccharides, and RtxA toxin.^[4–6]^ The overall mortality rate for patients infected with *V vulnificus* can reach up to 35%, with a further increase in mortality rates as treatment is delayed.^[7,8]^ Clinically, patients often have a history of contact with aquatic products, including seafood like marine fish, freshwater fish, crabs, shrimp, and mollusks. Many patients miss the optimal treatment window due to a lack of recognition of the severity of the condition or inadequate clinical experience at the treating hospital, leading to rapid deterioration of the condition. This can result in fatal complications such as sepsis, septic shock, necrotizing fasciitis, and limb necrosis, ultimately resulting in death or lifelong disability.^[9,10]^

*V vulnificus* is a Gram-negative bacterium belonging to the Vibrio genus and the Vibrionaceae family, is halophilic, anaerobic, and primarily widespread in seawater.^[11,12]^ It is also known as the marine *V vulnificus* or marine Vibrio. Humans and animals can be infected through wounds or the gastrointestinal tract. In recent years, clinical research on *V vulnificus* infections has predominantly been case reports, with rare multi-case analysis studies.^[13–15]^ Therefore, the purpose of this study is to conduct a retrospective descriptive analysis of 14 patients with confirmed *V vulnificus* infection caused by aquatic product-related trauma, admitted between 2020 and 2023. By summarizing their clinical characteristics, laboratory findings, diagnostic methods, treatments, and outcomes, we aim to propose a practical and efficient diagnostic and treatment protocol. This protocol is intended to improve early recognition and diagnostic and treatment of the infection, reduce misdiagnosis and delayed treatment, and ultimately lower mortality and disability rates.

## 2. Materials and methods

### 2.1. General information of patients

Retrospective analysis of 14 patients (including marine and freshwater fish, crabs, etc) with *V vulnificus* infections due to traumatic exposure to aquatic products admitted to our department from January 2020 to December 2023. This study was approved by the Ethics Committee of Zhongshan People’s Hospital (Approval No. ZS2020-11). All procedures involving human participants were conducted in accordance with the Declaration of Helsinki. Informed consent was obtained from all patients or, where necessary, from their legal guardians prior to inclusion in the study, particularly regarding the use of clinical data and images for research and publication purposes.

*Inclusion criteria*: Patients were included if they had an acute disease course, defined as symptom onset within 72 hours following trauma related to aquatic products, and a confirmed *V vulnificus* infection. Confirmation was based on either positive microbial culture from wound secretions, tissue, or blood samples, or identification via metagenomic next-generation sequencing (mNGS). Additionally, only patients with complete medical records and at least one valid follow-up (either in-person or by telephone) within 6 months after discharge were included. There were no restrictions on age, gender, or preexisting medical conditions.

*Exclusion criteria*: Patients were excluded if their disease course exceeded 72 hours before hospital admission, if *V vulnificus* infection could not be confirmed by laboratory methods, or if they demonstrated poor compliance with treatment, such as refusing recommended procedures or being discharged against medical advice within 48 hours. Patients who were lost to follow-up or declined follow-up contact were also excluded.

### 2.2. Therapeutic approaches

Based on the patient’s medical history, symptoms, signs, and auxiliary examination results, and combined with previous treatment experience, all patients with aquatic product-related trauma received early combined antibiotic therapy for diagnostic anti-infective treatment upon admission. The surgery timing was determined decisively, with rapid progression and good compliance as criteria. The surgical procedure involved: first, making an incision along the skin lines or in an S-shaped pattern within the area of skin redness and swelling, opening the fascia to the muscular layer, achieving thorough decompression, and performing debridement with hydrogen peroxide, povidone–iodine, and sterile saline. Medical pulse lavage devices were commonly used during this process. The wound was left open and treated with iodine gauze, vaseline gauze, or vacuum sealing drainage. Second, once the infection was under control and new granulation tissue appeared, the wound was repaired using sutures, skin grafting, or flap transfer techniques. Then, for patients with uncontrollable infection, ischemic necrosis in fingers/toes/limbs, and rapidly deteriorating local or systemic conditions, prompt amputation was performed when necessary.

For patients who refused surgery, ample and course-appropriate sensitive antibiotics were used for anti-infective and supportive treatment, with close monitoring of the limb and systemic progression.

### 2.3. Observational indicators and the establishment of a diagnostic and treatment protocol for acute *V vulnificus* infection

We collected and summarized patient information, including gender, age, injury and onset time, admission time, injury cause, affected limb, initial symptoms, and signs, laboratory findings upon admission and during hospitalization, comorbidities, complications, time from admission to first surgery, treatment plan, number of surgeries, microbiological diagnosis method and time, outcome and prognosis, and length of hospital stay. We analyzed these data to compare hospital stay, age, and time from admission to first surgery between patients with varying degrees of limb infection, those who survived with intact limbs versus those who died or underwent amputation, and the time to diagnosis using different microbiological methods. For surviving patients without amputation, the Brunnstrom scale was used at the last follow-up to assess hand function recovery. The scale divides hand function into stages I to VI, with I to II classified as disabled, III to IV as incomplete disability, and V to VI as functional recovery, with higher stages indicating better function. An activities of daily living (ADL) score above 60 suggests the patient can largely manage ADL independently. Scores between 60 and 40 indicate the need for assistance, 40 to 20 suggest a high level of assistance is required, and below 20 indicates complete dependence. By integrating the medical history, symptoms, signs, and treatment process of *V vulnificus* infections, we established a rapid diagnostic and treatment protocol for acute *V vulnificus* infections resulting from aquatic product-related trauma.

### 2.4. Statistical methods

Data analysis was performed using SPSS 29.0 statistical software (New York). For continuous data that followed a normal distribution, mean and standard deviation were reported. For data that did not follow a normal distribution, a median with interquartile range (M [Q1, Q3]) was used. Given the small sample size, the analysis focused on descriptive statistics to illustrate clinical patterns. Limited exploratory comparisons were made for illustrative purposes only, without formal hypothesis testing.

## 3. Results

### 3.1. General information

Of the patients, there were 8 males and 6 females; their ages ranged from 25 to 75 years, with a mean of (58.43 ± 14.41) years. Four patients were affected on the right limb, and 10 on the left. Three cases were reported from March to May, 2 from June to August, 8 from September to November, and one from December to the following February. Two patients were admitted 2 days post-injury, and 12 within 1 day. Symptoms developed within 2 to 24 hours after injury (mean: 13.11 ± 6.61 hours). Eight out of the 14 patients had underlying diseases, including type 2 diabetes, coronary heart disease, hyperlipidemia, hypertension, pulmonary infection, cerebrovascular disease, hepatitis B carrier, post-valve replacement, post-gastrointestinal stromal tumor surgery, gout, kidney stones, gallstones, liver cysts, liver nodules, renal atrophy, and cholecystitis. All patients were followed up, with the last follow-up ranging from 4 to 1496 days, averaging (674.36 ± 455.61) days.

### 3.2. Clinical characteristics of patients with *V vulnificus* infection at admission

**Figure 1.**
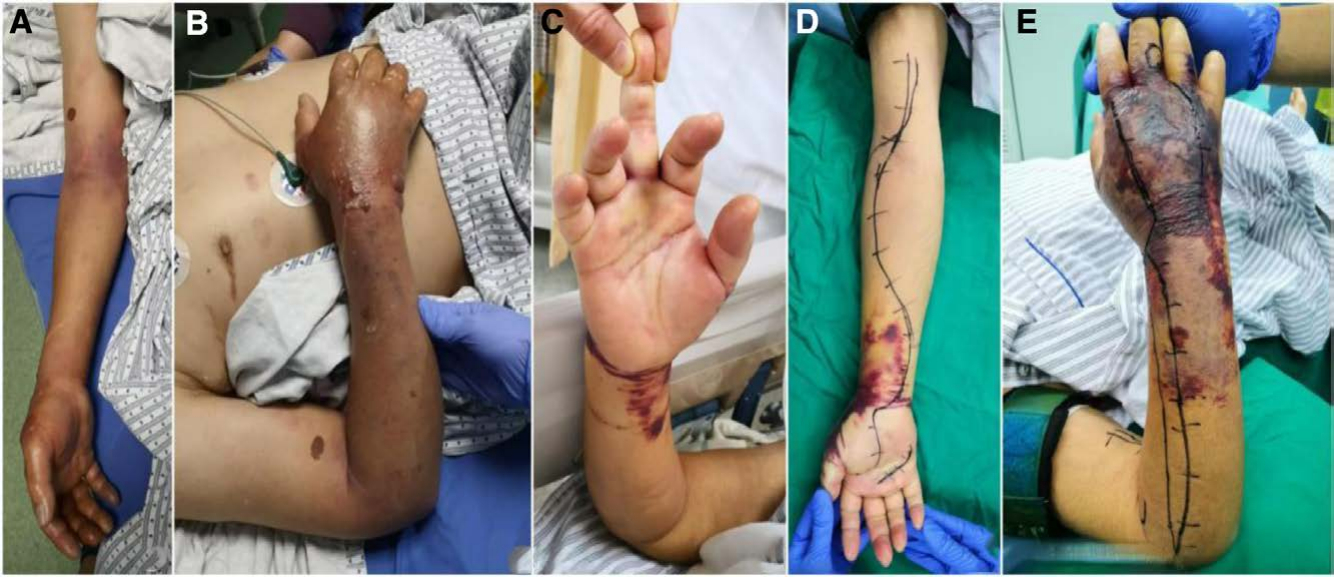
Typical clinical manifestations of limb trauma after *Vibrio vulnificus* infection. (A) and (B) The same patient, showing redness and tenderness from the fingers to above the elbow with multiple blisters, widespread increased skin tension, pain with passive finger extension, poor peripheral blood flow in the fingers, diminished sensation, and a barely palpable radial artery pulse, consistent with the presentation of compartment syndrome. The patient died within 4 days of admission. (C) to (E) A different patient with an initial punctate skin lesion near the palmar aspect of the right middle finger metacarpophalangeal joint. The dorsal hand and mid-distal forearm exhibit subcutaneous hemorrhage, ecchymosis, and large areas of epidermal necrolysis, with the lesion extending above the elbow joint.

All patients had a history of trauma associated with aquatic products, including 8 cases of marine fish punctures, 3 cases of punctures from unspecified fish species, 2 cases of crab punctures, and 1 case of freshwater fish puncture. The initial site of injury was most commonly the hand, with 6 cases involving the palm area, 6 cases affecting a single finger, 1 case involving the wrist, and 1 case of the distal thigh. In 7 cases, the lesion extended from the hand to the forearm upon admission; in 2 cases, it extended from the hand to above the elbow; in 2 cases, it extended from the hand to the wrist; in 2 cases, it extended from the fingers to the palm; and in 1 case, the lesion was confined to the distal thigh. Two patients presented with hypotension (<90/60 mm Hg); 4 with fever (≥37.3 °C), with temperatures ranging from 37.4 °C to 39.3 °C; and 6 with tachycardia (≥100 beats/min), with heart rates between 102 and 134 beats/min. All patients had significant redness and swelling in the affected limb, which could spread proximally or peripherally within hours. Initial injury sites exhibited punctate or laceration-like skin lesions. The skin over the swollen area was warm to the touch, tender, and had increased tension. There was a painful limitation of movement at the finger, wrist, or knee joints. Six cases showed petechiae and subcutaneous hemorrhage; 6 had poor peripheral blood flow and sensation in the affected limb; 5 experienced pain with passive finger extension; 3 had blisters; and 1 had epidermal necrolysis. A typical clinical presentation can be seen in Fig. 1.

### 3.3. Laboratory findings at admission for patients with *V vulnificus* infection

In 12 patients, the white blood cell count (WBC, ×10^9^/L) was elevated, while 1 had a count below the normal range and 1 had a normal count, with an average of 15.44 (9.76, 20.65) × 10^9^/L. Five patients had a low hemoglobin concentration (HGB, g/L), with the others within normal limits, averaging 133.07 ± 24.81 g/L. Three patients had a low platelet count (PLT, ×10^9^/L), with the rest normal, averaging 128.71 ± 52.77 × 10^9^/L. Six patients had a low sodium level (Na, mmol/L), with the rest normal, averaging 136.93 ± 3.05 mmol/L. Four patients had a low potassium level (K, mmol/L), with the rest normal, averaging 3.62 ± 0.49 mmol/L. Seven patients had a prolonged prothrombin time (PT, S), with the others normal, averaging 13.10 (11.98, 14.78) S. Seven patients also had a prolonged prothrombin time ratio, with the others normal, averaging 1.15 (1.06, 1.32). The activated partial thromboplastin time (APTT, S) was 28.90 (25.50, 34.58) S, with 2 cases above the normal range. Five patients had low albumin (Alb, g/L), with the others normal, averaging 41.84 ± 4.00 g/L. Five patients had elevated aspartate aminotransferase (AST, U/L), 1 had a low level, and the rest were normal, averaging 30.43 ± 15.64 U/L. Four patients had elevated alanine aminotransferase (ALT, U/L), with the rest normal, averaging 19.00 (14.75, 31.75) U/L. Six patients had elevated creatinine (Cr, μmol/L), with the rest normal, averaging 95.00 (79.50, 138.25) μmol/L. All 14 patients had an elevated procalcitonin (PCT, ng/mL), averaging 6.86 (2.13, 16.60) ng/mL. Twelve patients had an elevated C-reactive protein (CRP, mg/L), with the rest normal, averaging 40.55 (9.86, 103.77) mg/L.

### 3.4. Diagnosis of *V vulnificus* infection

Upon admission, wound secretions from all 14 patients were sent for laboratory microbiological culture, yielding 10 positives for *V vulnificus* and 4 negatives. The positive cultures (10/14) were sensitive to levofloxacin, ciprofloxacin, meropenem, imipenem, ceftazidime, cefepime, gentamicin, ampicillin, ampicillin/sulbactam, piperacillin, piperacillin/tazobactam, cefoperazone/sulbactam, amikacin, and co-trimoxazole. The turnaround time from specimen collection to culture result was (1.68 ± 0.63) days. Wound secretion samples from 3 patients and 1 blood sample from another patient were sent for mNGS detection, all of which identified *V vulnificus*. The turnaround time for mNGS from sample receipt to result was 1 day for all samples. There was no statistically significant difference in the turnaround time between the 2 detection methods (*Z* = ‐1.718, *P* = .086). The time from admission to diagnosis was (2.71 ± 1.56) days. The time to diagnosis was longer in patients who underwent multiple negative cultures before confirmation via mNGS, as observed in this case series.

### 3.5. Treatment and prognosis for patients with *V vulnificus* infection upon admission

**Figure 2.**
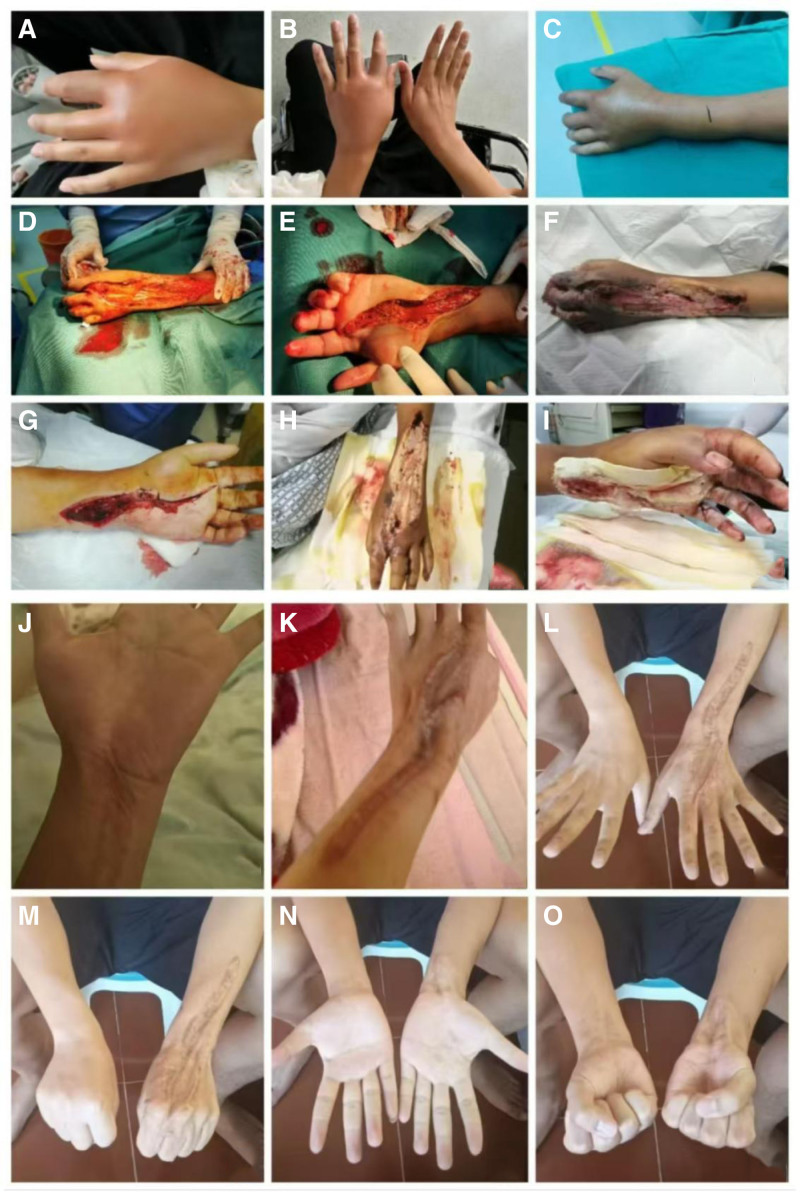
A typical case 1 of the treatment process and prognosis. (A) and (B) The affected limb at admission, showing marked redness, elevated skin temperature, and tenderness in the web space and surrounding areas of the left hand when compared to the unaffected (right) limb. (C) The rapid progression of swelling proximally to the forearm. (D) and (E) The surgical incision and debridement of the affected limb, which included the palm, dorsal and palmar aspects of the forearm, and mid-distal segments. (F) and (G) The wound status on the third postoperative day. (H) and (I) The wound status on the fifth postoperative day. (J) and (K) The healed wound after 6 months postoperatively. (L) to (O) The functional recovery of the affected limb compared to the unaffected limb after 1 year, achieving Brunnstrom stage VI.

**Figure 3.**
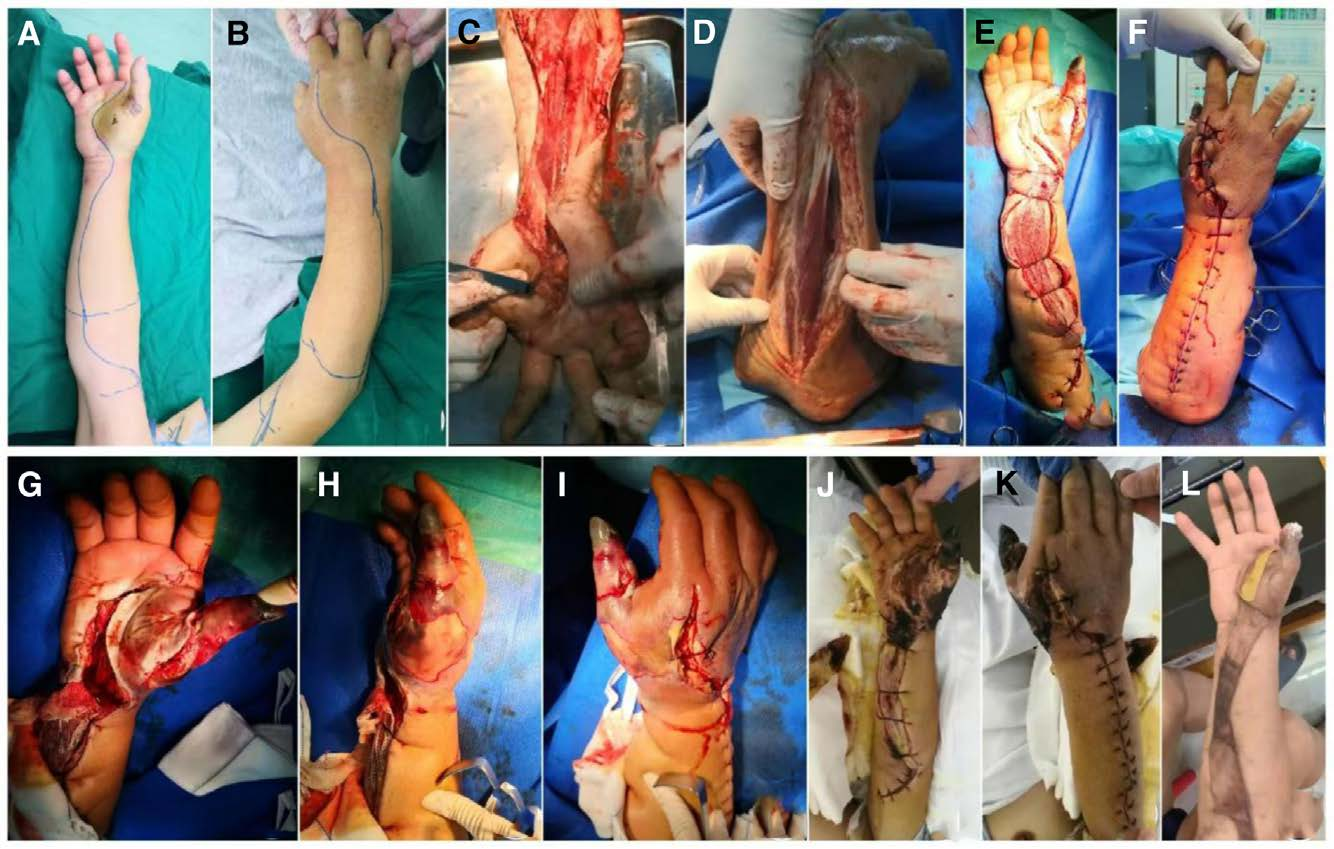
A typical case 2 of the treatment process and prognosis. Upon admission, a punctate skin lesion was observed near the volar aspect of the right thumb metacarpophalangeal joint, with redness, elevated skin temperature, severe tenderness, and increased skin tension in the palm, dorsal hand, and mid-distal forearm, as shown in (A) and (B). During surgery, extensive decompressive incisions were made on the thumb, palm, dorsal, and palmar aspects of the forearm, and mid-distal segments, as depicted in (C) and (D). Postoperative wound care was performed routinely, and on the ninth postoperative day, partial debridement and suture of the wound were performed after stabilization, as shown in (E) and (F), with ischemic necrosis of the right thumb evident in (G) to (I). After the second surgery, aggressive wound care was continued, and the demarcation of the necrotic area became clearer, as seen in (J) and (K). On days 23 and 30 after the second surgery, the patient underwent debridement and autologous free skin grafting with xenogeneic skin coverage on the upper right limb, and amputation of the right thumb with autologous free skin grafting from the upper arm, respectively. By 6 months postoperatively, the wound had largely healed, with a Brunnstrom stage IV functional recovery, as shown in (L).

All patients received early empiric antibiotic therapy with cephalosporins (cefoperazone, cefotaxime, ceftriaxone, ceftazidime, and cefuroxime), piperacillin/tazobactam, or imipenem, combined with levofloxacin, ciprofloxacin, moxifloxacin, or doxycycline, and underwent incision and debridement surgery as soon as possible. During hospitalization, 11 patients developed various complications, including sepsis (6 cases), limb/digit/toe necrosis (6 cases), infectious/septic shock (5 cases), compartment syndrome in the affected limb (5 cases), multiple organ dysfunction syndrome (MODS) (4 cases), pulmonary infection (4 cases), acute kidney injury (2 cases), pleural effusion (2 cases), arrhythmias (2 cases), and gastrointestinal bleeding (2 cases). Nine patients required intensive care unit treatment, and 6 received venous-venous hemodialysis continuous renal replacement therapy. Two patients died during hospitalization: one due to sepsis, septic shock, severe pneumonia, atrial fibrillation, MODS (acute cardiac insufficiency, acute liver failure), and gastrointestinal bleeding; and another due to septic shock and MODS involving the lungs, heart, liver, kidneys, and hematological system. Six patients underwent amputation/digit/toe surgery (6/14). At the last follow-up, based on the Brunnstrom staging, 7 patients showed hand function recovery, 2 had incomplete disability, and 1 had severe disability. According to the ADL score, all surviving patients were capable of self-care to a basic extent. Patients with lesions extending across multiple joints tended to have longer hospital stays, based on observed clinical trends in this series. There was no significant difference in hospital stay between patients who survived with intact limbs (42.50 ± 18.12 days) and those who died or underwent amputation/digit/toe surgery (54.83 ± 45.86 days), *P* = .498. There was also no significant difference in age (years) between the 2 groups (54.00 ± 17.97 for survivors with intact limbs and 64.33 ± 3.88 for those who died or underwent amputation/digit/toe surgery), *P* = .154. The time from admission to the first surgical procedure was not significantly different between the groups (1.75 [1.44, 6.13] hours for survivors with intact limbs and 1.80 [1.50, 2.00] hours for those who died or underwent amputation/digit/toe surgery), *Z* = ‐0.066, *P* = .947. Typical treatment cases are shown in Figs. 2 and 3.

### 3.6. Diagnostic and treatment protocol for limb *V vulnificus* infection via aquatic product-related trauma

#### 3.6.1. Comprehensive patient assessment

Before admission, thoroughly inquire about the type of aquatic product causing the injury and the onset of symptoms. It is preferable to retain the aquatic product or its images for reference. Clarify the patient’s symptoms and signs, understand their medical history and comorbidities, and document the extent of the limb lesion with corresponding timestamps.

#### 3.6.2. Implementation of the RiCH scoring system

The RiCH scoring system, proposed by Hong Guangliang (16), rapidly identifies and filters out high-risk patients for *V vulnificus* infection. It assesses 3 key criteria: (1) rapid progression of limb lesions, (2) the presence of hypotension, and (3) a history of alcoholism, chronic liver disease, or immunocompromised status. Each criterion scores one point. A score of ≥2 indicates potential for *V vulnificus* sepsis, necessitating immediate initiation of rescue protocols. Clinically, more emphasis is placed on criteria (1) and (2).

#### 3.6.3. Preliminary pathogen sampling and rapid testing

For patients with punctate skin lesions or small, dry wounds where it is challenging to directly obtain wound secretions, blood samples should be promptly taken for mNGS testing and blood culture.

In cases of significant limb edema with less apparent skin lesions, fluid from the edemtous area should be aspirated for mNGS and microbiological culture.

For patients with extensive exposed wounds, blisters, or hematomas, wound secretions, blister fluid, hematoma fluid, or lesion tissue samples should be collected for microbiological culture, mNGS testing, or histopathological examination. Initial pathogen sampling is ideally completed before the administration of antibiotics or surgical disinfection.

#### 3.6.4. Early use of sensitive antibiotics

For patients with a clear history of aquatic product injury, typical symptoms of limb erythema, swelling, heat, and pain, and a high suspicion of *V vulnificus* infection, it is recommended to initiate early, adequate, and extended-course combination antibiotic therapy. This should include intravenous administration of piperacillin/tazobactam or cefoperazone/sulbactam, third-generation cephalosporins, in combination with levofloxacin or ciprofloxacin, quinolone antibiotics, for diagnostic anti-infective treatment of at least 7 to 10 days. Antibiotic therapy should be adjusted based on the results of pathogen detection.

#### 3.6.5. Comprehensive preoperative preparation and timely surgery

Early surgical incision, decompression, and thorough debridement are recommended for patients with rapidly progressive limb swelling, elevated skin temperature, skin tension, and severe local pain. It is advisable to collect multiple tissue and secretion samples from the wound for bacterial culture and mNGS testing before debridement, if feasible, and also for histopathological examination. The initial lesion site should be thoroughly decompressed and debrided during surgery. The wound can be covered with vacuum sealing drainage or left open with iodine-soaked gauze, vaseline gauze, or burn gauze dressing.

## 4. Discussion

*V vulnificus* is an opportunistic pathogen commonly found in marine, lacustrine, and riverine environments.^[16–18]^ It can invade the human body through open wounds or via the orifices of the face, leading to infections.^[19]^ All patients in this study were infected by *V vulnificus* through open wounds, with a history of being punctured or cut by aquatic products during purchase or preparation. The initial injury site is often inconspicuous, and patients often fail to take the condition seriously, leading to rapid disease progression. The incidence of *V vulnificus* infections is more common from April to November, with fewer cases during the relatively cold winter months. This is likely related to the bacterium’s preference for warmer temperatures.^[20,21]^ In this study, 12 of the 14 patients presented with symptoms during this period, which aligns with most literature reports. Eight cases occurred from September to November, indicating that autumn is a peak period for *V vulnificus* infections. Most of the patients in this study were middle-aged or elderly, with an average age of 58.43 ± 14.41 years. This demographic may be at higher risk due to more frequent exposure to aquatic products through daily cooking and purchasing activities. The majority of patients sought medical attention within 1 day of injury. However, 2 patients presented 2 days after their injury, both of whom experienced severe outcomes: one required amputation and died, while the other had the longest hospital stay after amputation.

Patients with chronic underlying conditions such as chronic liver disease, diabetes, renal failure, autoimmune diseases, and Mediterranean anemia are susceptible to *V vulnificus* infections and often develop severe complications.^[22–24]^ In this study, one deceased patient was a hepatitis B carrier with cirrhosis and post-mitral valve replacement. The patient had not been on long-term oral anticoagulation before admission, and had significantly elevated levels of WBC, PT, prothrombin time ratio, ferritin (Ferr), N-terminal pro-brain natriuretic peptide, PCT, and CRP upon admission, with progressive increases. Conversely, levels of HGB, PLT, serum iron (Fe), and transferrin were markedly decreased and continued to decline. The patient received venous-venous hemodialysis continuous renal replacement therapy and underwent 3 surgeries: 2 for incision and debridement of the affected limb with decompression, and the last as a shoulder disarticulation. Unfortunately, the patient died of MODS and septic shock within 4 days. The rapid progression of the condition, which was difficult to control, may have been associated with Fe overload due to chronic liver disease. Literature suggests that a ferruginous environment in the host’s serum can accelerate the replication of *V vulnificus*.^[25–27]^ The patient’s Ferr levels increased from 1317 ng/mL to 6730 ng/mL, while the initial Fe level was only 2 μmol/L (normal range 11–30 μmol/L), indicating that the free Fe in the serum was continuously consumed by bacteria, and the Fe overload environment facilitated the growth of *V vulnificus*. Among the 14 patients in this study, 7 had their serum Fe levels tested at admission, with 6 showing significantly lower levels than normal, ranging from 0 to 9 μmol/L (average 3 ± 3.16 μmol/L), and 1 normal. Ten patients had Ferr levels tested during hospitalization, with 3 showing significantly elevated levels, one reaching up to 6730 ng/mL; all 3 of these patients developed MODS, one died, one experienced prolonged bone marrow suppression after discharge, and one had the longest hospital stay and underwent shoulder disarticulation surgery. These observations suggest a potential association between elevated Ferr levels and more severe clinical outcomes in *Vibrio vulnificus* infection. This highlights the importance of promptly assessing serum Fe, transferrin, and Ferr levels, although further studies with larger cohorts are warranted to validate this finding. However, not all patients in this study underwent these tests during their hospital stay, which may be due to the varying degrees of recognition, understanding, and clinical experience of the treating physicians regarding this condition.

Laboratory indicators following *Vibrio vulnificus* infection may show elevations in WBC, PCT, CRP, PT, APTT, AST, ALT, and Cr, exceeding the normal range. Conversely, levels of HGB, PLT, Na, and Alb may be below normal. However, there are cases where WBC may be normal or even below normal at admission, which warrants high vigilance. For instance, in this study, a patient with a normal WBC count at admission saw it gradually drop to 2.33 × 10^9^/L, below the normal lower limit, and then rise to 21.09 × 10^9^/L by the last measurement. This patient ultimately died after only 4 days of hospitalization. Another patient with a low WBC count at admission (2.65 × 10^9^/L) further decreased to 2.37 × 10^9^/L, rose after crossing the normal range to a peak of 43.90 × 10^9^/L, and then decreased to 9.94 × 10^9^/L by the last measurement, still above the normal range. This patient had the longest hospital stay and, despite surviving, underwent amputation due to worsening limb infection and necrosis.

In this study, all 12 patients had HGB levels below normal in their final tests. This may be related to chronic blood loss from the limb lesion, consumption due to severe infection, blood loss during surgery, and the hemolytic and cytotoxic activities of toxins such as *V vulnificus* hemolysin and the cell lysis activity of phospholipase secreted by the bacterium.^[28,29]^ Further research is needed to understand these mechanisms. *V vulnificus* infection can cause thrombocytopenia and prolongation of PT and APTT, leading to a tendency for bleeding and the appearance of subcutaneous ecchymosis and petechiae.^[30]^ One patient in this study had significantly prolonged PT and APTT values of 63 S and 51.4 S, respectively. This patient had a history of mitral and aortic valve replacement surgery and was on long-term anticoagulation with warfarin. This, combined with *V vulnificus* infection, may have contributed to the marked prolongation of PT and APTT. The patient experienced gastrointestinal bleeding and ultimately died from sepsis, septic shock, and MODS, suggesting that long-term preoperative anticoagulation may be a risk factor for fatal outcomes in *V vulnificus* infection. Elevations in AST, ALT, and Cr, as well as decreased Alb, indicate that *V vulnificus* can cause liver and kidney damage, as well as hypoalbuminemia. In this study, all 14 patients had significantly elevated PCT levels, and 12 had elevated CRP levels. These early and significant increases in inflammatory markers such as PCT and CRP are crucial for the early judgment and assessment of acute severe infections.

In recent years, the consensus among literature reports is that patients with *V vulnificus* infections should be treated with early, sufficient, and extended courses of combination antibiotics, often recommending third-generation cephalosporins/piperacillin combined with quinolones.^[31]^ This study followed this principle with its 14 patients. Based on experience, multiple antibiotics were used before pathogen detection results were available, and these were confirmed to be sensitive to *V vulnificus* in microbial culture sensitivity tests. This is crucial, even decisive, for early infection control. In addition to effective antibiotic use, timing surgery accurately is also a critical step. *V vulnificus* infections often have a rapid onset and progression, which can be difficult to control with medication alone. Timely surgical incisions for decompression and thorough debridement of the wound are more effective and direct. Both treatments should complement each other without bias. The diagnosis of *V vulnificus* is usually achieved through microbial culture, but there are instances of negative results. Relying solely on culture can delay diagnosis and treatment. mNGS addresses this issue by rapidly sequencing nucleic acid samples and comparing them with genomic sequences to identify the types and proportions of microorganisms present. In this study, there was no significant difference in the time required for results between culture and mNGS. Early mNGS can significantly increase diagnosis efficiency. This indicates that, in clinical practice, with advancements in detection technology and improvements in critical care systems, both culture and mNGS can complete pathogen detection quickly, and their complementary use has become a trend.

In this study, upon admission, all patients exhibited varying degrees of infection lesions spreading proximally in the limbs. The study categorized these lesions into 2 groups based on the extent of spread: those confined or extending across one joint, and those extending across 2 or more joints. Patients in the latter group had longer hospital stays (70.4 ± 41.80 days) compared to the former (35.22 ± 17.28 days), based on observed clinical trends in this case series. This suggests that delayed treatment allows bacteria to rapidly proliferate and invade tissue spaces, causing extensive soft tissue infections and potentially bloodstream infections, sepsis, and septicemia. This progression from localized to systemic infection increases treatment complexity and mortality risk, resulting in substantial healthcare costs. The aggressive nature of *V vulnificus* infection may be attributed to the bacterium’s production of various virulence factors, including exotoxins (such as *V vulnificus* hemolysin, multifunctional autoprocessing repeat toxin, *V vulnificus* protease, and phospholipase) and capsular polysaccharides.^[32,33]^ Literature has reported that three-quarters of *V vulnificus* infection patients die within 2 days due to MODS, highlighting MODS as a critical factor in mortality.^[34]^ In this study, 4 patients developed MODS (4/14), with 2 fatalities. One patient had the longest hospital stay of 137 days and ultimately required amputation, while another experienced prolonged pancytopenia post-discharge (manifested by WBC levels persistently below the normal range and difficult to correct). These cases demonstrate that MODS in *V vulnificus* infection patients leads to a rapid decline in systemic immunity, resistance, and multi-organ function, posing a high mortality risk and poor prognosis.

*V vulnificus* infections are relatively rare in clinical practice, but there has been a trend towards increased incidence due to changing climates and environmental conditions.^[35,36]^ In recent years, the authors’ department has treated numerous patients injured by various aquatic products who were ultimately not diagnosed with *V vulnificus*. Some patients opted for conservative treatment, while others actively underwent surgery. However, all these patients were treated as highly suspected cases of *V vulnificus* infection, following the appropriate diagnostic and treatment protocols, and ultimately experienced favorable outcomes.

This study focuses on a relatively small sample size of only 14 confirmed cases of *V vulnificus* infection. Due to this limitation, the study is primarily descriptive and lacks the statistical power to support formal case–control or cohort analyses. Additionally, the data were collected from a single center, which may limit the generalizability of the findings to other geographic regions or clinical settings. The retrospective nature of the study also introduces potential sources of bias, such as incomplete documentation and unmeasured confounders. Moreover, the absence of a control group makes it difficult to directly compare the effectiveness of different treatment strategies or diagnostic approaches. These limitations should be taken into account when interpreting the results, and future prospective, multicenter studies with larger sample sizes and control groups are warranted to validate our observations and proposed protocol.

## 5. Conclusions

This study underscores the critical importance of early diagnosis and treatment in *V vulnificus* infections associated with aquatic product-related injuries, given the high mortality and morbidity rates observed. The diagnostic and treatment protocol developed in this research provides clinicians with a robust framework for enhancing the efficiency and accuracy of early intervention. Notably, the application of mNGS has significantly expedited diagnostic processes, highlighting the pivotal role of molecular technologies in the context of antibiotic treatment strategies. Our clinical observations support the integration of advanced diagnostic tools into the diagnostic and treatment of *V vulnificus* infections and emphasize the ongoing need for research into antibiotic resistance to ensure the continued efficacy of treatment approaches. This protocol stands as a cornerstone in the effective diagnostic and treatment of *V vulnificus* infections and underscores the importance of further investigation into the intersection of molecular diagnostics and antibiotic stewardship.

## Acknowledgments

The authors would like to express their gratitude to the Department of Orthopaedics at Zhongshan City People’s Hospital for the support and facilities provided throughout the study. The participants are also sincerely thanked for their invaluable participation and contributions. The research was conducted without external funding or additional support.

## Author contributions

**Conceptualization:** Donghua Ma, Jinjun Wang, Baoying Fan, Qing Liu, Zhiyong He.

**Data curation:** Donghua Ma, Jinjun Wang, Baoying Fan.

**Formal analysis:** Donghua Ma, Baoying Fan, Jianji Liang, Zhiyong He.

**Funding acquisition:** Jianji Liang.

**Investigation:** Donghua Ma, Jinjun Wang, Baoying Fan, Jianji Liang, Qing Liu, Zhiyong He.

**Methodology:** Donghua Ma, Jinjun Wang, Baoying Fan, Jianji Liang, Qing Liu, Zhiyong He.

**Software:** Zhiyong He.

**Supervision:** Donghua Ma, Jinjun Wang, Baoying Fan, Qing Liu, Zhiyong He.

**Validation:** Donghua Ma, Jinjun Wang, Baoying Fan, Qing Liu, Zhiyong He.

**Visualization:** Donghua Ma, Jinjun Wang, Baoying Fan, Qing Liu.

**Writing – original draft:** Donghua Ma, Zhiyong He.

**Writing – review & editing:** Donghua Ma, Zhiyong He.
